# Genomic Insights into Adaptations of Trimethylamine-Utilizing Methanogens to Diverse Habitats, Including the Human Gut

**DOI:** 10.1128/mSystems.00939-20

**Published:** 2021-02-09

**Authors:** Jacobo de la Cuesta-Zuluaga, Tim D. Spector, Nicholas D. Youngblut, Ruth E. Ley

**Affiliations:** a Department of Microbiome Science, Max Planck Institute for Developmental Biology, Tübingen, Germany; b Department of Twin Research and Genetic Epidemiology, King’s College London, London, UK; Vanderbilt University

**Keywords:** *Methanomassiliicoccales*, archaea, comparative genomics, human gut, metagenomes, microbiome

## Abstract

*Archaea* of the order Methanomassiliicoccales use methylated amines such as trimethylamine as the substrates for methanogenesis. They form two large phylogenetic clades and reside in diverse environments, from soil to the human gut. Two genera, one from each clade, inhabit the human gut: Methanomassiliicoccus, which has one cultured representative, and “*Candidatus* Methanomethylophilus,” which has none. Questions remain regarding their distribution across biomes and human populations, their association with other taxa in the gut, and whether host genetics correlate with their abundance. To gain insight into the *Methanomassiliicoccales* clade, particularly its human-associated members, we performed a genomic comparison of 72 *Methanomassiliicoccales* genomes and assessed their presence in metagenomes derived from the human gut (*n* = 4,472, representing 22 populations), nonhuman animal gut (*n* = 145), and nonhost environments (*n* = 160). Our analyses showed that all taxa are generalists; they were detected in animal gut and environmental samples. We confirmed two large clades, one enriched in the gut and the other enriched in the environment, with notable exceptions. Genomic adaptations to the gut include genome reduction and genes involved in the shikimate pathway and bile resistance. Genomic adaptations differed by clade, not habitat preference, indicating convergent evolution between the clades. In the human gut, the relative abundance of *Methanomassiliicoccales* spp. correlated with trimethylamine-producing bacteria and was unrelated to host genotype. Our results shed light on the microbial ecology of this group and may help guide *Methanomassiliicoccales*-based strategies for trimethylamine mitigation in cardiovascular disease.

**IMPORTANCE** *Methanomassiliicoccales* are less-known members of the human gut archaeome. Members of this order use methylated amines, including trimethylamine, in methane production. This group has only one cultured representative; how its members adapted to inhabit the mammalian gut and how they interact with other microbes is largely unknown. Using bioinformatics methods applied to DNA from a wide range of samples, we profiled the abundances of these Archaea spp. in environmental and host-associated microbial communities. We observed two groups of *Methanomassiliicoccales*, one largely host associated and one largely found in environmental samples, with some exceptions. When host associated, these *Archaea* have smaller genomes and possess genes related to bile resistance and aromatic amino acid precursors. We did not detect *Methanomassiliicoccales* in all human populations tested, but when present, they were correlated with bacteria known to produce trimethylamine. Due to their metabolism of trimethylamine, these intriguing *Archaea* may form the basis of novel therapies for cardiovascular disease.

## Abstract (precis)

*Methanomassiliicoccales* are less-known members of the human gut archaeome. Members of this order use methylated amines, including trimethylamine, in methane production.

## INTRODUCTION

Archaea spp. generally make up a tenth or less of the biomass of the human gut microbiota; however, they are widely prevalent and occupy a unique metabolic niche, utilizing byproducts of bacterial metabolism as the substrates for methanogenesis (1). Members of Methanobacteriales are the dominant species of the human gut archaeome (1, 2). These include Methanobrevibacter smithii, which uses CO_2_, formate and H_2_ as the substrates for methane production (3), and Methanosphaera stadtmanae, which consumes methanol and H_2_ (4). Through methanogenesis, *Archaea* decrease partial pressures of H_2_, potentially increasing the energetic efficiency of primary fermenters and the production of short-chain fatty acids (5).

A second archaeal lineage, the order *Methanomassiliicoccales*, is also found within the human gut, yet its members are less well characterized than those of *Methanobacteriales*. Members of this order, including human-derived Methanomassiliicoccus luminyensis, *“Candidatus* Methanomassiliicoccus intestinalis,” and “*Candidatus* Methanomethylophilus alvus,” perform H_2_-dependent methylotrophic methanogenesis for their sole energy source (6–8). Their genomes encode several methyltransferases and associated proteins that reduce methylamines, methanol, and methylated sulfides to methane (9). Studies based on 16S rRNA and *mcrA* gene diversity analysis indicate that the order *Methanomassiliicoccales* is made up of two large clades, which mostly group species that have either a free-living (FL) or host-associated (HA) lifestyle (10, 11). Based on analyses of the genomes from three human-derived species from both clades, Borrel et al. (9) suggested that each clade colonized the mammalian gut independently. Members of the HA clade, including the human-associated “*Ca*. M. alvus,” might be expected to show adaptations similar to those of other methanogens from the gut microbiota (12, 13). How members of the FL clade, including the human-associated M. luminyensis and *“Ca*. M. intestinalis,” have converged on the gut niche remains to be explored.

A better understanding of the ecology of *Methanomassiliicoccales* may be of interest to human health, as they can utilize mono-, di-, and trimethylamine (TMA) as the substrates for methanogenesis in the gut (14). TMA, a byproduct of bacterial metabolism of carnitine, choline, and other compounds, is transformed in the liver into trimethylamine *N*-oxide (TMAO) (15). Circulating TMAO inhibits cholesterol transport and promotes its accumulation in macrophages, inducing the formation of atherosclerotic plaques (16). Decreasing TMA levels in the gut and reducing circulating TMAO levels have been proposed as a therapeutic strategy for cardiovascular disease (17). One way to use the gut microbiome to this end would be to boost levels of *Methanomassiliicoccales* (18). To accomplish this goal requires a deeper understanding of its ecology.

Here, we conducted a comparative analysis of 71 *Methanomassiliicoccales* genomes, together with an additional metagenome-assembled genome (MAG) corresponding to a strain of “*Ca*. M. alvus” that we retrieved by metagenome assembly of gut samples from subjects of the United Kingdom Adult Twin Registry (TwinsUK) cohort (19). We used 305 publicly available metagenomes to assess the prevalence of taxa across various habitat types. While the two large clades grouping host-associated (HA) and free-living (FL) taxa are generally enriched in host-associated and environmental metagenomes, a few exceptions stand out. Our results showed that the repertoire of adhesion proteins encoded by the genomes of taxa from each clade tended to differ. Genes involved in bile resistance and the shikimate pathway are likely involved in the adaptation to the gut environment of members of the HA clade, but not for the FL clade. Thus, gut-adapted members converged on life in the gut using different genomic adaptations. *Methanomassiliicoccales* genera present in the human gut positively correlate with TMA-producing bacteria.

## RESULTS

### Genome-based phylogeny confirms two large *Methanomassiliicoccales* clades.

**FIG 1:**
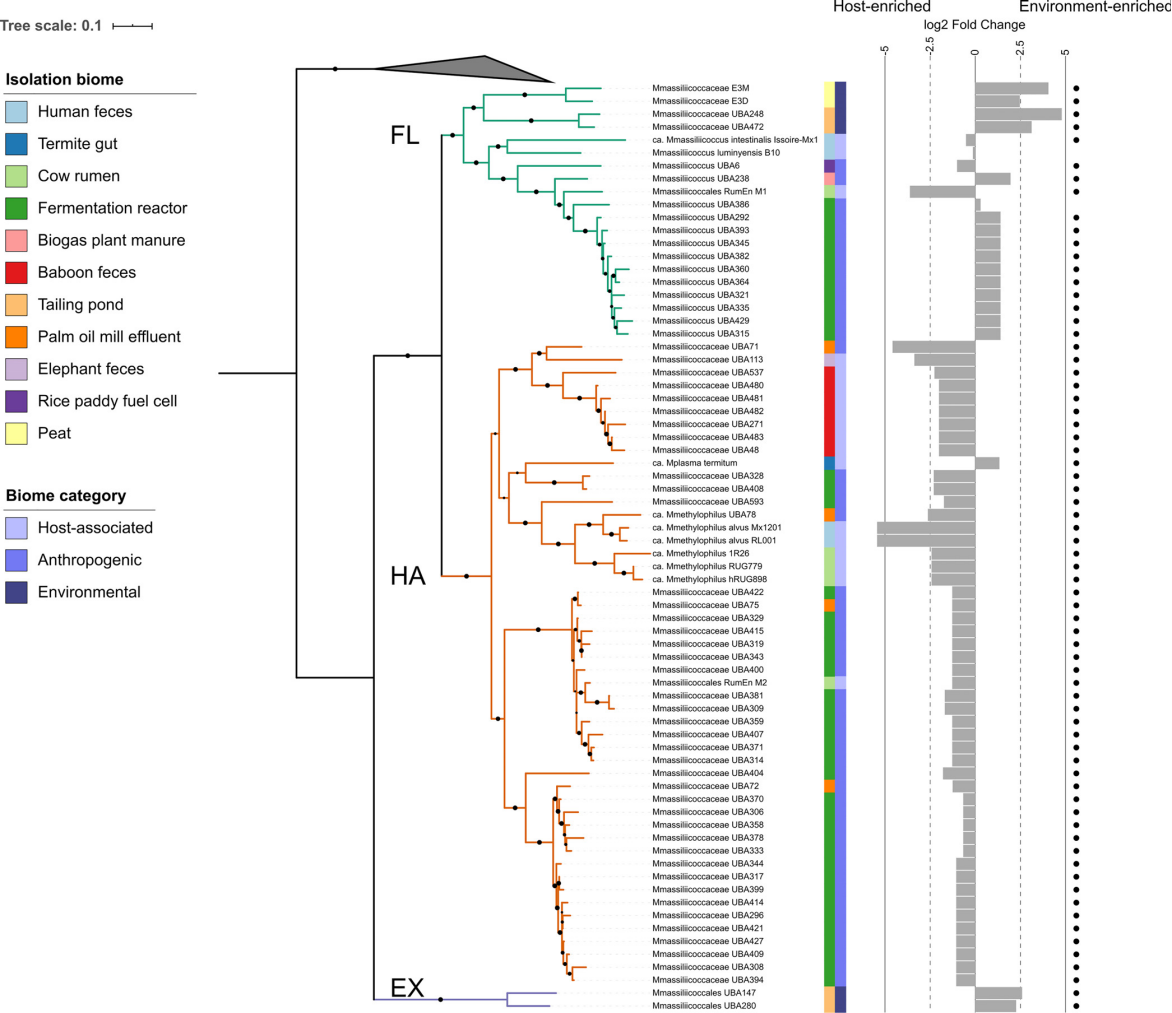
The order Methanomassiliicoccales forms two large clades that loosely follow the source of isolation. Maximum-likelihood phylogeny of concatenated single-copy marker genes. The gray triangle corresponds to Thermoplasma acidophilum, Picrophilus oshimae, Ferroplasma acidarmanus, Acidiplasma aeolicum, and Cuniculiplasma divulgatum, which are outgroup taxa from class Thermoplasmata. Black circles indicate bootstrap values of >80 (of 1,000 bootstrap permutations), branch color represents the clade, and the scale bar represents the number of amino acid substitutions per site. Colored strips show the source of isolation of each of the included genomes and the general category to which the source belongs. Bar plots show the genome abundance enrichment in gut metagenome samples compared to environmental samples, calculated using DESeq2; dots indicate taxa with significant enrichment in either host or environmental biomes (adj. *P* < 0.05). Mmassiliicoccaceae, *Methanomassiliicocceae*; Mmassiliicoccus, *Methanomassiliicoccus*; Mplasma, *Methanoplasma*; Mmethylophilus, *Methanomethylophilus*; Mmassiliicoccales, *Methanomassiliicoccales*.

Based on whole-genome phylogenetic analysis, the order *Methanomassiliicoccales* forms two clades with robust support (Fig. 1). This phylogeny is in agreement with previously reported phylogenies based on 16S rRNA and *mcrA* genes (10, 20, 21). A third distal clade was formed by two closely related MAGs generated in a recent massive metagenome assembly effort (22), which we labeled external (EX) (Fig. 1). We use the terminology of Borrel et al. (23), as follows: the clade including *Methanomassiliicoccus* is labeled free living (FL), and the clade containing “*Candidatus* Methanomethylophilus” is labeled host associated (HA).

As observed previously (10), the reported source of the genomes was not always consistent with the clade in which it was grouped. For instance, while publicly available genomes originally retrieved from human, baboon, elephant, and cow gastrointestinal tracts were related to “*Candidatus* Methanomethylophilus” (HA), this clade also contained MAGs derived from digestors and reactors (Fig. 1) reportedly not treating animal waste (see Table S1A in the supplemental material). Moreover, MAGs retrieved from pit mud of solid-state fermentation reactors used for the production of Chinese liquor were present in both the HA and FL clades (Table S1A). Similarly, “*Ca*. M. intestinalis” Issoire-Mx1, *M. luminyensis* B10, and *Methanomassiliicoccales* archaeon RumEn M1, all retrieved from mammal hosts, grouped in the FL clade.

10.1128/mSystems.00939-20.7 TABLE S1 (A) NCBI assembly accession number, genome characteristics, study information, and source of isolation of 71 publicly available genomes from the order *Methanomassiliicoccales* retrieved from NCBI in June 2018, plus the “Ca. M. alvus” metagenome-assembled genome (MAG) reported here. Study accession and title of UBA genomes obtained from supplementary tables of Parks et al., 2017 (https://doi.org//10.1038/s41564-017-0012-7) (22), otherwise, obtained from the NCBI BioProject database. (B) SRA and MGnify accession information of publicly available metagenome samples from gastrointestinal and environmental biomes. (C) SRA, study, and country information of publicly available human gut metagenome samples. (D) Prevalence and mean abundance of “*Candidatus* Methanomethylophilus” and *Methanomassiliicoccus* taxa across multiple human populations. (E) InterPro, eggNOG, and Prokka annotations of gene clusters significantly enriched in clade FL compared to clade HA. (F) Coabundance and cooccurrence measures of positively associated taxa of the human gut microbiota with “*Ca*. Methanomethylophilus” and *Methanomassiliicoccus*. For each of the two *Methanomassiliicoccales* genera, the observed cooccurrences, expected cooccurrences, adjusted *P* values of cooccurrence, and association of abundances across samples (rho) are provided. (G) Intraclass correlation coefficients (ICC) and adjusted *P* values of relative abundances of *Methanomassiliicoccus* and other control taxa in monozygotic and dizygotic twins. Download Table S1, XLSX file, 0.2 MB. Copyright © 2021 de la Cuesta-Zuluaga et al. 2021 de la Cuesta-Zuluaga et al. This content is distributed under the terms of the Creative Commons Attribution 4.0 International license.

### Abundance of *Methanomassiliicoccales* clades differs in gastrointestinal and environmental samples.

We assessed the abundance of species-level representative *Methanomassiliicoccales* taxa in publicly available metagenomes that included 145 samples from gastrointestinal tracts of nonhuman animals, such as cats, pigs, elks, cows, mice, white-throated woodrats, trout, chickens, and geese, and 160 environmental samples from sediment, ice, and diverse water and soil sources (Table S1B).

**FIG 2:**
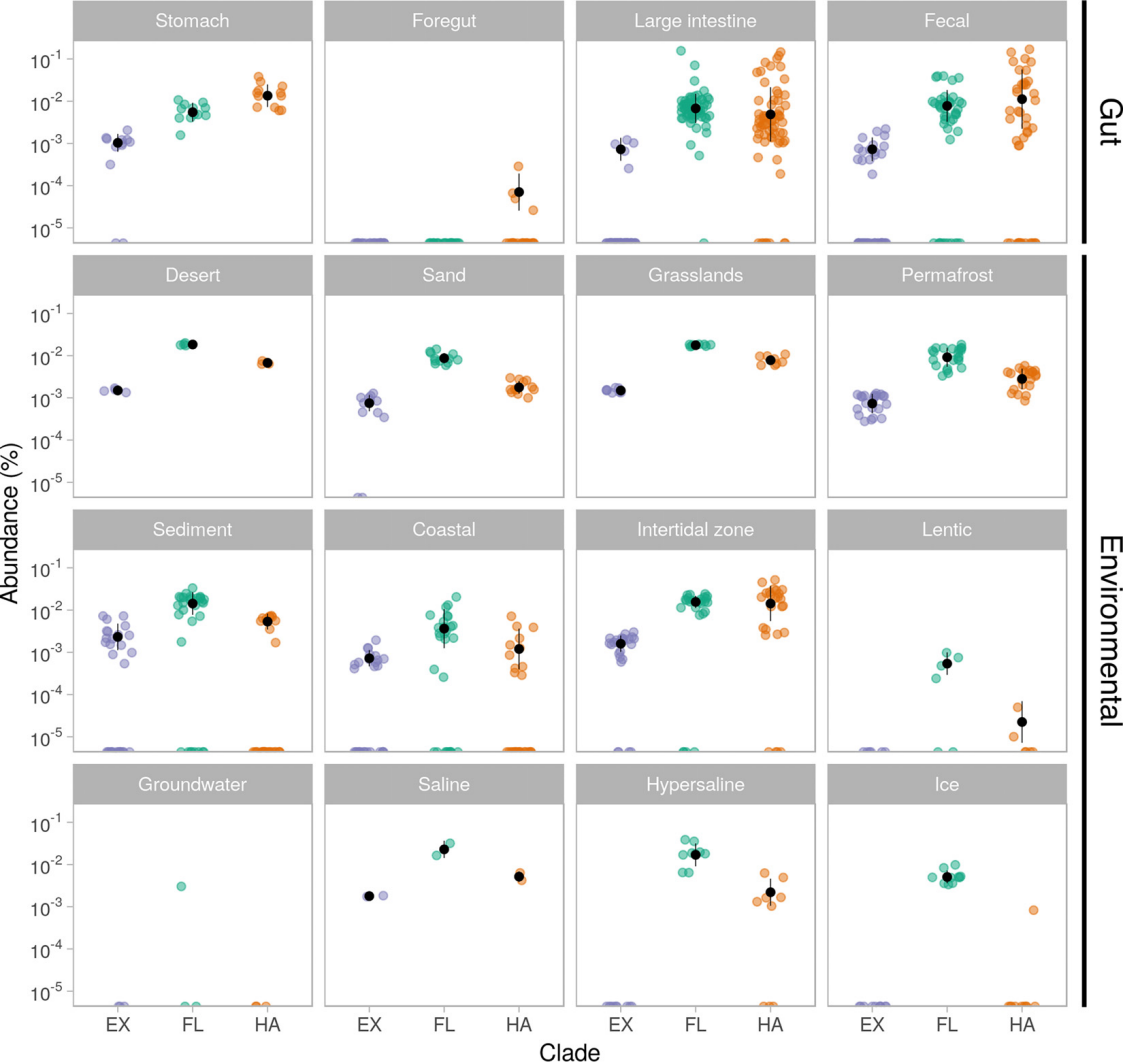
*Methanomassiliicoccales* clades are widespread but not abundant across a range of environments and animal hosts. Combined abundance of representative genomes of the EX (purple), FL (green), and HA (orange) clades in metagenome samples from diverse biomes, as follows: stomach (*n* = 12), foregut (*n* = 23), large intestine (*n* = 66), fecal (*n* = 44), desert (*n* = 4), sand (*n* = 12), grasslands (*n* = 8), permafrost (*n* = 22), sediment (*n* = 31), coastal (*n* = 28), intertidal zone (*n* = 25), lentic (*n* = 6), groundwater (*n* = 3), saline (*n* = 2), hypersaline (*n* = 9), and ice (*n* = 10). Abundances were calculated for individual genomes using KrakenUniq and aggregated by clade. The *y* axis is in logarithmic scale; black points indicate mean relative abundance in percentage, and black bars indicate standard deviation.

Taxa from all three clades were detected in a wide range of metagenomes from environmental and gut origin. We observed differences in environmental preference by clade. Abundance of taxa from clade EX was highest in environmental metagenomes (0.001% ± 0.0012%) (Fig. 1). These were also detected in gut samples (0.0002% ± 0.0005%), albeit with a very low abundance, and in in fecal (0.0003% ± 0.0005), large intestine (0.0001% ± 0.0002%), and stomach (0.0009% ± 0.0006%) metagenomes (Fig. 2). Given their low abundances, further analysis is focused on the FL and HA clades.

The aggregated abundance of clades FL and HA differed across biomes (Fig. 2). In agreement with their names, HA clade members were more abundant in host-associated samples, and FL in non-host-associated samples (Fig. 2). The prevalence and abundance of *Methanomassiliicoccales* taxa varied across animal hosts, yet the overall abundance patterns were consistent across hosts and sample types (see Fig. S2 in the supplemental material).

The combined abundance of members of clade FL was higher in samples from environmental biomes (0.01% ± 0.008%), although nonzero abundances were observed in digestive system metagenomes (0.008% ± 0.015%), with some samples containing levels comparable to that of clade HA (Fig. 2).

The mean abundance of clade HA in aggregate was higher in metagenomes from gut samples (0.014% ± 0.03%) compared to those from environmental biomes (0.004% ± 0.008%). However, among the environmental biomes, nonzero abundances of clade HA were detected in freshwater (0.002% ± 0.003%), marine (0.006% ± 0.011%), saline and alkaline (0.002% ± 0.002%), and soil (0.004% ± 0.003%) samples.

We further validated the differences in clade abundances across biomes by generating a dendrogram of *Methanomassiliicoccales* taxa using the DESeq2-based log fold change of individual taxa on gut versus environmental biomes (i.e., the effect size of the test as a measure of enrichment on a given environment). We then compared the structure of this dendrogram with that of the phylogenomic tree and found that they were positively correlated (cophenetic correlation = 0.67; *P* < 0.01).

Overall, we observed a low abundance of individual *Methanomassiliicoccales* taxa across all samples, ranging from 0 to 0.15% (Fig. 2 and Fig. S1). Their prevalence across hosts differed; they were prevalent in animals such as elks, pigs, poultry, and cattle, while in others, such as trout and geese, they were largely absent (see Fig. S3 in the supplemental material). The enrichment analysis of individual taxa from clade FL from diverse biomes showed that while most were significantly enriched in environmental metagenomes (adjusted [adj.] *P* < 0.1), some taxa showed the opposite enrichment. *M. luminyensis* and Methanomassiliicoccus sp. UBA386 were not significantly enriched in gut or environmental biomes. “*Ca*. M. intestinalis” Issoire-Mx1, Methanomassiliicoccales archaeon RumEn M1, and *Methanomassiliicoccus* sp. UBA6 were significantly enriched in gut biomes (Fig. 1), although they were also present in multiple environmental biomes (fig. S1).

10.1128/mSystems.00939-20.2 FIG S1 *Methanomassiliicoccales* taxa from all clades are widespread but not abundant across a range of environments and animal hosts. The abundances of members of the free-living (FL) and host-associated (HA) clades are comparable within similar biomes, particularly in animal-derived metagenomes. Abundance of each representative genome on diverse metagenome and environmental metagenome samples colored by clade (green, FL; orange, HA; purple, external [EX]). Abundances calculated for individual genomes using KrakenUniq and aggregated by clade. Note that the *y* axis is in logarithmic scale and each plot has a different scale. Black points indicate mean relative abundance in percentage, and black bars indicate standard deviation. Metagenome samples from stomach (*n* = 12), foregut (*n* = 23), large intestine (*n* = 66), fecal (*n* = 44), desert (*n* = 4), sand (*n* = 12), grasslands (*n* = 8), permafrost (*n* = 22), sediment (*n* = 31), coastal (*n* = 28), intertidal zone (*n* = 25), lentic (*n* = 6), groundwater (*n* = 3), saline (*n* = 2), hypersaline (*n* = 9), and ice (*n* = 10) biomes. Download FIG S1, TIF file, 2.0 MB. Copyright © 2021 de la Cuesta-Zuluaga et al. 2021 de la Cuesta-Zuluaga et al. This content is distributed under the terms of the Creative Commons Attribution 4.0 International license.

10.1128/mSystems.00939-20.3 FIG S2 Abundance of *Methanomassiliicoccales* clades varies across animal hosts and sample types. Samples from the same host but different sampling sites show consistent patterns. Metagenome samples from cat (*n* = 30), pig (*n* = 30), rainbow trout (*n* = 23), mouse (*n* = 16), poultry (*n* = 14), goose (*n* = 12), cattle (*n* = 8), white-throated woodrat (*n* = 8), and Eurasian elk (*n* = 3). See Fig. S1 for details. Download FIG S2, TIF file, 0.5 MB. Copyright © 2021 de la Cuesta-Zuluaga et al. 2021 de la Cuesta-Zuluaga et al. This content is distributed under the terms of the Creative Commons Attribution 4.0 International license.

10.1128/mSystems.00939-20.4 FIG S3 Animal hosts show different patterns of prevalence of individual *Methanomassiliicoccales* taxa. cat (*n* = 30), pig (*n* = 30), rainbow trout (*n* = 23), mouse (*n* = 16), poultry (*n* = 14), goose (*n* = 12), cattle (*n* = 8), white-throated woodrat (*n* = 8), and Eurasian elk (*n* = 3). See Fig. S1 for details. Download FIG S3, TIF file, 2.2 MB. Copyright © 2021 de la Cuesta-Zuluaga et al. 2021 de la Cuesta-Zuluaga et al. This content is distributed under the terms of the Creative Commons Attribution 4.0 International license.

When assessed on a per-taxon basis, the vast majority of clade HA taxa were significantly enriched in gut samples (adj. *P* < 0.1), with the exception of “*Candidatus* Methanoplasma termitum.” which was highly abundant in soil samples from grasslands and water samples from intertidal zones (Fig. 1).

### Genome characteristics and core genes functions differ between *Methanomassiliicoccales* clades.

Given the tendency of clades FL and HA to be enriched in environmental or animal metagenomes, respectively, we searched for genes and genome features linked to putative adaptations of *Methanomassiliicoccales* to an animal gut. For this, we compared 72 genomes from *Methanomassiliicoccales* taxa retrieved from humans, nonhuman animals, and environmental sources.

**FIG 3:**
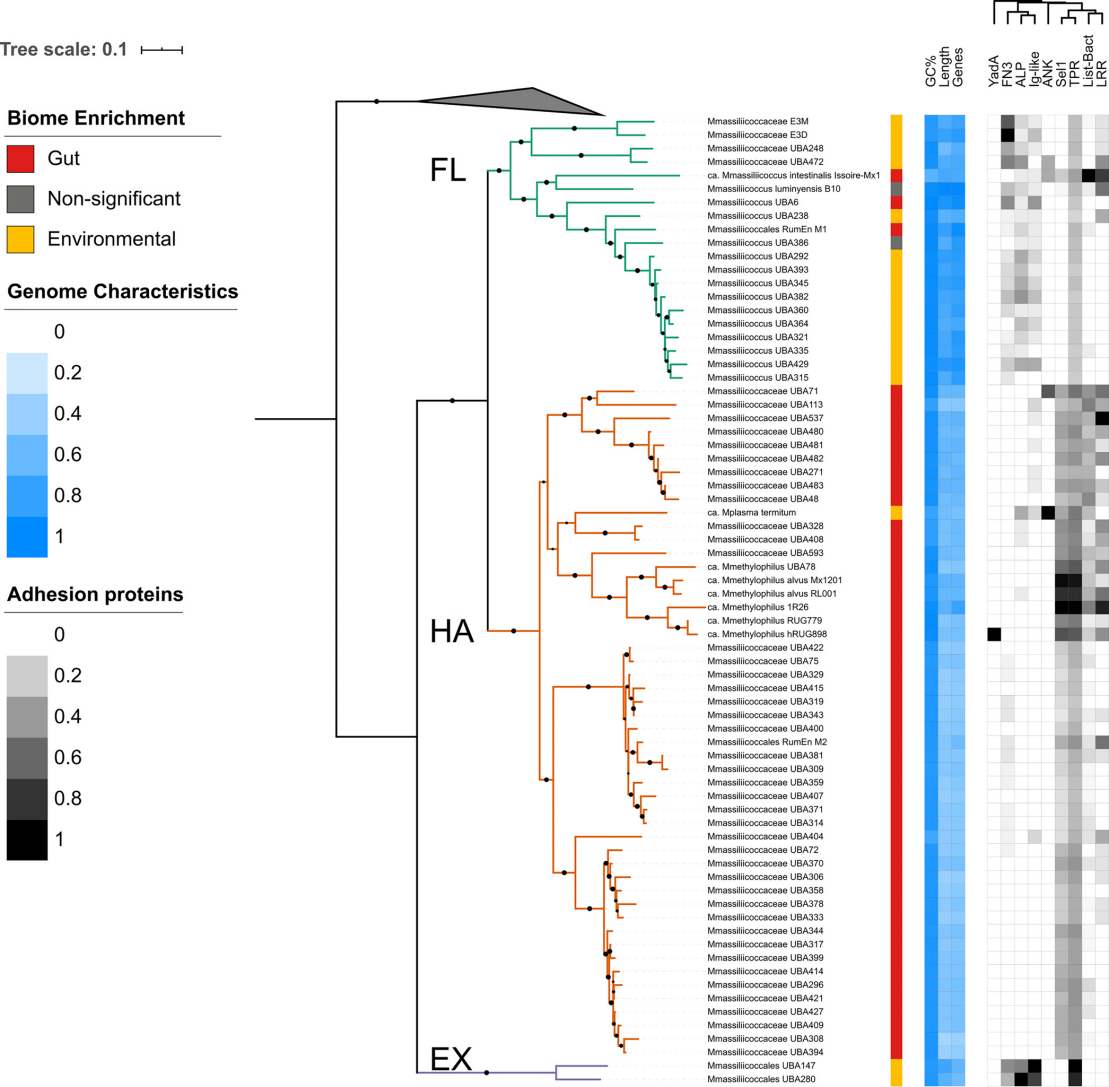
Genome characteristics and adhesion proteins of *Methanomassiliicoccales* reflect division of the order into clades. Note that members of clade FL not enriched in environmental biomes resemble those of clade HA. The phylogeny is the same as that shown in Fig. 1. The colored strip summarizes the biome enrichment analysis. Heatmaps show genome features, including genome GC content (“GC%,” range, 41.26% to 62.74%), genome length (“Length,” 969.311 bp to 2.620.233 bp), and number of predicted genes (“Genes,” 1,057 to 2,607) (blue scale), or a repertoire of eukaryote-like proteins: YadA-like domain (YadA; 0, 1), fibronectin type III (FN3; 0, 20) domains, bacterial Ig-like domains (Ig-like; 0, 12), ankyrin repeats (ANK; 0, 3), Sel1-containing proteins (Sel1; 0, 29), tetratricopeptide repeats (TPR; 7, 40), *Listeria-Bacteroides* repeat-containing proteins (List-Bact; 0, 26), and leucine-rich repeats (LRR; 0, 9) (gray scale shows columns ordered by hierarchical clustering); and adhesion-like proteins (ALP; 0, 12). On both heatmaps, the color intensity of each feature is relative to the maximum value of each category.

We observed that genomes were more similar to others closely located on the phylogeny in terms of genome GC content, genome length and total gene count (local indicator of phylogenetic association [LIPA] adj. *P* < 0.01 in all cases) (Fig. 3). To determine whether these features differed between clades, while accounting for the autocorrelation due to evolutionary history, we performed a phylogenetic analysis of variance (ANOVA). Clade FL taxa had significantly larger genomes (mean ± standard deviation [SD], 1,985.1 ± 245.1 kb) than either clade HA (1,318.3 ± 187.3 kb) or clade EX (1,872.2 ± 173.8 kb) (phylogenetic ANOVA adj. *P* = 0.028). In accordance with this, clade FL also had the highest gene count (FL, 2,153.1 ± 233.7 genes; HA, 1,377.7 ± 187.7 genes; EX, 1,567.0 ± 90.5 genes; adj. *P* = 0.025). While this was nonsignificant, clades HA and EX taxa tended to have a lower GC content than clade FL taxa (FL, 59.1% ± 4.8%; HA, 55.8% ± 2.8%; EX, 54.4% ± 0.5%; adj. *P* = 0.6).

To compare gene presence and absence across clades, we performed a pangenome analysis. After identification of orthologous gene clusters based on sequence similarity using panX software, we obtained 13,695 clusters, of which 7,312 were present at least once in clade FL, 6,592 in clade HA, and 1,833 in clade EX. A large proportion of gene clusters were of unknown function according to the clusters of orthologous genes (COG) functional classification (38.4% ± 4.3%); gene clusters of unknown function tended to be detected in one or two genomes (see Fig. S4A in the supplemental material).

10.1128/mSystems.00939-20.5 FIG S4 Small clusters of unknown function dominate the pangenome of the order *Methanomassiliicoccales*. Gene cluster frequency spectrum of the order *Methanomassiliicoccales* separated by (A) unknown or (B) known function; the *x* axis represents the number of genomes that have at least one gene in a given cluster, and the *y* axis is the frequency of clusters of a given size. Note the difference in scale of the *y* axis between panels A and B. (C) Fraction of gene clusters belonging to each clusters of orthologous genes (COG) category per clade. Core clusters were defined as being present in ≥80% of genomes of a clade; for the complete order (i.e., all included *Methanomassiliicoccales* taxa without grouping them by clade), gene clusters were present in ≥80% of the included genomes and in at least one member of each clade. The proportion of clusters of unknown functions in the core genome of each clade was large and varied between clades, ranging from 23.0% in clade HA to 38.5% in clade EX. The proportion of unknown clusters was lowest in the complete taxonomic order, where it only accounted for 14.7% of gene clusters. COG functional classification descriptions by groups, as follows. Information storage and processing: B, chromatin structure and dynamic; J, translation, ribosomal structure, and biogenesis; K, transcription; L, replication, recombination, and repair. Cellular processes and signaling: D, cell cycle control, cell division, and chromosome partitioning; M, cell wall/membrane/envelope biogenesis; N, cell motility; O, posttranslational modification, protein turnover, and chaperone; T, signal transduction mechanisms; U, intracellular trafficking, secretion, and vesicular transport; V, defense mechanisms; Z, cytoskeleton. Metabolism: C, energy production and conversion; E, amino acid transport and metabolism; F, nucleotide transport and metabolism; G carbohydrate transport and metabolism; H, coenzyme transport and metabolism; I, lipid transport and metabolism; P, inorganic ion transport and metabolism; Q, secondary metabolites biosynthesis, transport, and catabolism. Poorly characterized: X, no annotation retrieved; S, function unknown. Download FIG S4, TIF file, 0.5 MB. Copyright © 2021 de la Cuesta-Zuluaga et al. 2021 de la Cuesta-Zuluaga et al. This content is distributed under the terms of the Creative Commons Attribution 4.0 International license.

**FIG 4:**
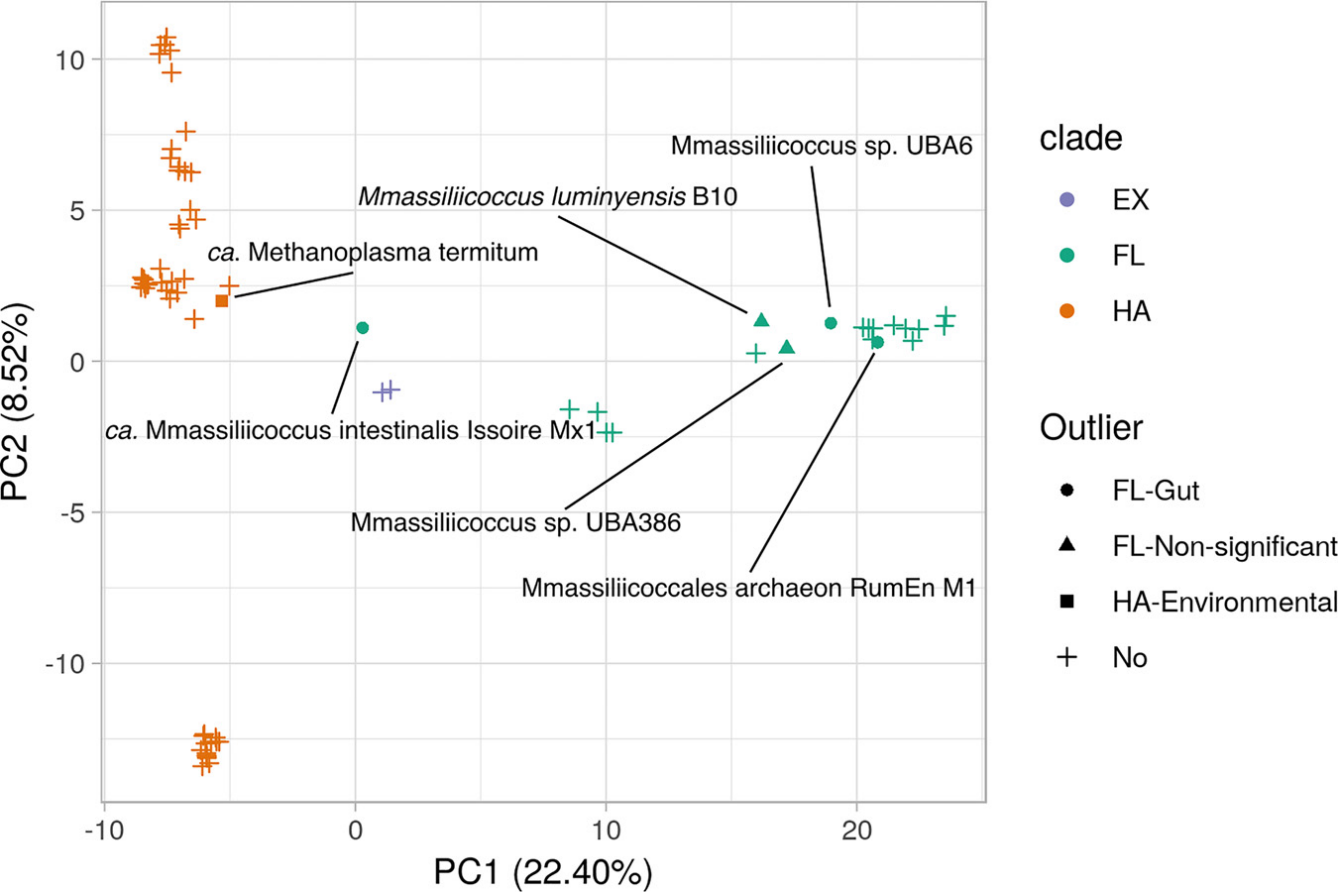
Ordination of gene content of *Methanomassiliicoccales* group taxa by phylogenetic clade rather than by biome enrichment. Principal-component analysis of the gene cluster presence of taxa from clades FL (green), HA (orange), and EX (purple). Highlighted points correspond to outliers, namely, taxa either not significantly enriched in environmental or gut biomes or with enrichment opposite to expectation given their clade.

Principal component analysis (PCA) of gene cluster presence/absence differentiated clades along principal component 1 (PC1) (Fig. 4). We defined outlier taxa as FL taxa enriched in gut biomes (*Methanomassiliicoccales* archaeon RumEn M1, *Methanomassiliicoccus* sp. UBA6, *“Ca*. M. intestinalis” Issoire-Mx1, *M. luminyensis* B10, and *Methanomassiliicoccus* sp. UBA386) and the HA taxon enriched in non-host biomes (“*Ca*. M. termitum”). Outliers mostly clustered with their close relatives, not with the taxa enriched in the same biome (Fig. 4), with the exception of *“Ca*. M. intestinalis” Issoire-Mx1, which did not cluster with either clade.

### Gene clusters enriched in clade HA evidence adaptation to the gut environment.

Because of the small number of genomes that cluster within clade EX, and because these are largely absent from animal-associated samples, subsequent analyses focus on comparisons between clades FL and HA.

To identify gene clusters potentially involved in the adaptation of members of Clade HA to a host environment, we compared the gene cluster content between clades. The gene cluster frequency spectrum shows many clusters present in few genomes; 7,990 (58.3%) gene clusters were singletons, and 2,002 (14.6%) were doubletons (Fig. S4A and B). After removing rare gene clusters by filtering those with near-zero variance, we included 2,937 clusters, which we then used to perform in phylogenetic ANOVA. Results reveal 14 gene clusters significantly enriched in HA compared to FL (adj. *P* < 0.1 in all cases) (Table 1). Three gene clusters are involved in detoxification and xenobiotic metabolism, namely, those encoding bile acid: sodium symporter, bleomycin resistance protein and HAD superfamily hydrolase. Two clusters are related to shikimate or chorismate metabolism, namely, those encoding chorismate mutase II and prephenate dehydratase. Other annotated clusters include the small unit of exonuclease VII, Holliday junction resolvase Hjc, nitrogen regulatory protein PII, xylose isomerase-like protein, and metal-binding domain containing protein; four had poor or no annotation (Table 1). Similar results were obtained when we performed this analysis without outlier taxa and when biome enrichment was used as independent variable (not shown), further indicating that genomic adaptations differ by clade, not habitat preference. Likewise, 89 clusters were enriched in clade FL compared to HA; these are presented in Table S1E.

**TABLE 1:** InterPro, eggNOG, and Prokka annotations of gene clusters significantly enriched in clade HA compared to clade FL

InterPro accession no.	InterPro annotation	NOG accession no.	COG category^*a*^	Prokka gene name	Prokka annotation
IPR002657	Bile acid:sodium symporter/arsenical resistance protein Acr3	COG0385@NOG	S		Hypothetical protein
IPR029068	Glyoxalase/bleomycin resistance protein/dihydroxybiphenyl dioxygenase	-	X		Hypothetical protein
IPR006357	HAD superfamily hydrolase, subfamily IIA	COG0647@NOG	G	*gph*	Glyceraldehyde 3-phosphate phosphatase
IPR002701	Chorismate mutase II, prokaryotic-type	COG1605@NOG	E	*aroQ*	Chorismate mutase
IPR001086	Prephenate dehydratase	COG0077@NOG	E	*pheA*	Prephenate dehydratase
IPR003761	Exonuclease VII, small subunit	COG1722@NOG	L	*xseB*	Exodeoxyribonuclease 7 small subunit
IPR002732	Holliday junction resolvase Hjc	COG1591@NOG	L	*rutD*	Putative aminoacrylate hydrolase RutD
IPR015867	Nitrogen regulatory protein PII/ATP phosphoribosyltransferase, C-terminal	COG3323@NOG	S		Hypothetical protein
IPR013022	Xylose isomerase-like, TIM barrel domain	11IHC@NOG	L		Hypothetical protein
IPR019271	Protein of unknown function DUF2284, metal-binding	11RTN@NOG	S		Hypothetical protein

a COG functional classification descriptions: E, amino acid transport and metabolism; L, replication, recombination, and repair; G, carbohydrate transport and metabolism; S, function unknown; X, no annotation retrieved.

### Genomic adaptations to the gut of members of the FL clade.

To determine whether outlier taxa belonging to clade FL had similar adaptations to the gut to those of members of clade HA, we explored gene clusters that were present in these outliers and in clade HA but were rare in other members of clade FL. We selected gene clusters present in the core genome of clade HA (i.e., present in at least 40 taxa or 80% of this clade, see Text S1 in the supplemental material) and present in less than half of the FL taxa. A total of 15 gene clusters were obtained, most of them encoded by only one of the outlier taxa. Two gene clusters, ferrous iron transport proteins A and B (InterPro accession numbers IPR030389 and IPR007167), were present in three of the outliers (*M. luminyensis*, *“Ca*. M. intestinalis” Issoire-Mx1, and *Methanomassiliicoccales* archaeon RumEn M1). Other clusters detected in more than one outlier included an uncharacterized membrane protein (InterPro number IPR005182, in *Methanomassiliicoccus* sp. UBA6 and *Methanomassiliicoccales* archaeon RumEn M1), a putative nickel-responsive regulator (InterPro number IPR014864, in *M. luminyensis* B10 and *Methanomassiliicoccus* sp. UBA386), and an ABC transporter (InterPro number IPR037294, in *M. luminyensis* B10 and *Methanomassiliicoccus* sp. UBA386). The remaining gene clusters, detected once, corresponded to transcriptional regulators or proteins of unknown function.

10.1128/mSystems.00939-20.1 TEXT S1 Supplementary methods contain detailed description of the tests carried in the present work and the versions of the software and R packages used. Supplementary results contain description of the retrieval of a genome from “*Candidatus* Methanomethylophilus alvus” by metagenomic assembly; comparison of core gene functions between *Methanomassiliicoccales* clades; and assessment of *Methanomassiliicoccales* with methanol producers in the human gut. Download Text S1, DOCX file, 0.02 MB. Copyright © 2021 de la Cuesta-Zuluaga et al. 2021 de la Cuesta-Zuluaga et al. This content is distributed under the terms of the Creative Commons Attribution 4.0 International license.

### The repertoire of adhesion proteins tended to differ between clades HA and FL.

We compared between FL and HA clades two large groups of membrane proteins involved in adhesion, namely eukaryote-like proteins (ELPs), a series of protein families involved in microbial adherence to the host (24), and adhesin-like proteins (ALPs), a class of proteins hypothesized to be involved in the microbe-microbe interactions of *Methanobacteriales* in the gut (13). We aggregated the counts of gene clusters annotated as the ALP and ELP classes and performed phylogenetic ANOVA. This analysis showed a trend toward differing repertoires of adhesion proteins by clade (Fig. 3), although we did not observe significant differences in the frequency of these factors (adj. *P* > 0.1 in all cases). Taxa from clade HA tended to have higher mean counts of tetratricopeptide repeats (TPR) (mean ± SD counts: HA, 16.30 ± 6.56, and FL. 9.55 ± 1.70), Sel1-containing repeats (Sel1) (HA, 9.32 ± 5.69, and FL, 0.35 ± 1.35), *Listeria-Bacteroides* repeats (List-Bact) (HA, 3.68 ± 3.76, and FL, 1.65 ± 5.78), and leucine-rich repeats (LRR) (HA, 1.5 ± 2.15, and FL, 1.1 ± 2.02) than FL taxa. Conversely, adhesin-like proteins (ALPs) (FL, 2.25 ± 1.48, and HA, 0.14 ± 0.61), Ig-like domains (FL, 1.55 ± 1.32, and HA, 0.20 ± 0.53) and fibronectin type III (FN3) domains (FL, 4.55 ± 5.09, and HA, 0.32 ± 0.62) tended to be more abundant in the genomes of members of clade FL. We did not detect invasion protein B (IalB) in any of the analyzed genomes.

Hierarchical clustering based on the presence or absence of adhesion factors largely grouped *Methanomassiliicoccales* taxa by clade (see Fig. S5 in the supplemental material). Additionally, all adhesion factors, with the exceptions of ankyrin repeats (ANK) and the *Yersinia* adhesin A-like domain (YadA), showed a significant phylogenetic signal (adj. *P* < 0.05 in all cases), further highlighting that closely related taxa had similar counts.

10.1128/mSystems.00939-20.6 FIG S5 The set of adhesion proteins tends to differ by clade. The tanglegram compares a dendrogram of adhesion genes calculated using hierarchical clustering of Jaccard index matrix (left) versus the maximum-likelihood phylogeny (right). Tip labels colored by clade: HA, orange; FL, green; EX, purple. Download FIG S5, TIF file, 0.9 MB. Copyright © 2021 de la Cuesta-Zuluaga et al. 2021 de la Cuesta-Zuluaga et al. This content is distributed under the terms of the Creative Commons Attribution 4.0 International license.

Interestingly, outlier taxa from clade FL had gene counts of several of the adhesion factors higher than the mean of their own clade and more characteristic of clade HA. In some cases, the gene counts were higher than the mean for clade HA. These included Listeria-Bacteroides repeats (gene cluster counts: *M. luminyensis*, 2; “*Ca*. M. intestinalis” Issoire-Mx1, 26; *Methanomassiliicoccales* archaeon RumEn M1, 2), Sel1 repeats (*M. luminyensis*, 1; “*Ca*. M. intestinalis” Issoire-Mx1, 6), and leucine-rich repeats (*M. luminyensis*, 5; “*Ca*. M. intestinalis” Issoire-Mx1, 7).

### *Methanomassiliicoccales* taxa cooccur with each other, with other *Archaea*, and with TMA-producing bacteria in the human gut.

**FIG 5:**
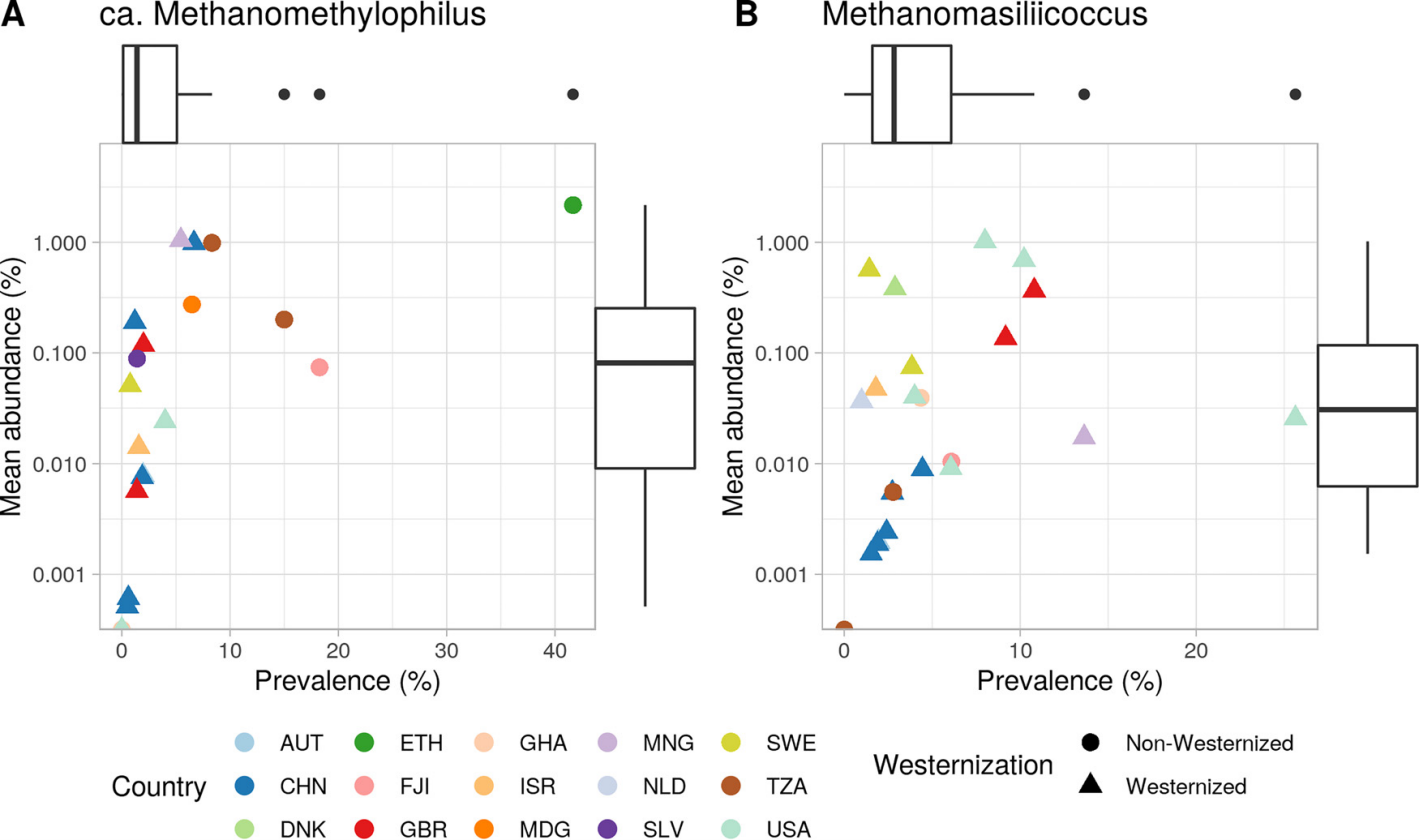
*Methanomassiliicoccales* are rare members of the human gut microbiota. Scatterplots of the genera (A) “*Ca*. Methanomethylophilus” and (B) *Methanomassiliicoccus* show that their prevalence and mean abundance is low across most studies and populations (35 data sets) with subjects (*n* = 4,472) from Austria (AUT), China (CHN), Denmark (DNK), Ethiopia (ETH), Fijo (FJI), Great Britain (GBR), Ghana (GHA), Israel (ISR), Madagascar (MDG), Mongolia (MNG), The Netherlands (NDL), El Salvador (SLV), Sweden (SWE), Tanzania (TZA), and the United States (USA).

We characterized the distribution of *Methanomassiliicoccales* spp. across a collection of human gut metagenomes derived from 34 studies. Together, the combined 4,472 samples represented people from 22 countries, resulting in 35 unique data sets (i.e., study-country combinations). Across the whole set, we detected just two genera, *Methanomassiliicoccus* (clade FL) and “*Ca*. Methanomethylophilus” (clade HA), both rare members of the human gut microbiota (Fig. 5). “*Ca*. Methanomethylophilus” was detectable in 19 out of 35 data sets; in these 19 data sets, it had a prevalence ranging from 0.5% to 41.7%, and mean abundance ranged from 4.8 × 10^−6^% to 2.2 × 10^−2^%. Similarly, *Methanomassiliicoccus* was detectable in 22 of the 35 data sets; in the 22 data sets, it had a prevalence range of 1% to 25.7% and a mean abundance range of 1.5 × 10^−5^% to 1.0 × 10^−2^% (Table S1D).

We tested associations of these two genera with age, sex, and Westernization status of the subjects using linear mixed models that included the data set and country as random effects. Subjects from non-Westernized countries had a significantly higher prevalence of “*Ca*. Methanomethylophilus” (mean prevalence ± SD: non-Westernized = 8.9% ± 28.5%, Westernized = 1.1% ± 10.3%; adj. *P* = 0.002). Westernized individuals were more likely to harbor higher prevalences of *Methanomassiliicoccus*, although differences were not significant (non-Westernized = 3.9% ± 19.4%, Westernized, 5.0% ± 21.7%; adj. *P* > 0.1). The age and sex of the individuals did not explain variance in the prevalence or abundance of either genus (adj. *P* > 0.1 in all cases).

To identify other microbial taxa positively associated with members of *Methanomassiliicoccales* in the human gut, we calculated a network of positively associated microorganisms (i.e., coabundant taxa) across samples (rho > 0.1 in all cases) (25). In addition, we determined which taxa were present with members of *Methanomassiliicoccales* at a greater prevalence than that expected by chance (i.e., cooccurring taxa) relative to a permuted null model (26). Results showed that both “*Ca*. Methanomethylophilus” and *Methanomassiliicoccus* were part of the same coabundance network, together with a third archaeal genus, Methanoculleus (order Methanomicrobiales). We did not find evidence of positive or negative abundance associations of either *Methanomassiliicoccales* genus with Methanobrevibacter. Coocurrence analysis showed a random association pattern between these taxa (*P* > 0.05 for both “*Ca*. Methanomethylophilus” and Methanomassiliicoccus), indicating that their ecological niches do not overlap that of Methanobrevibacter.

**FIG 6:**
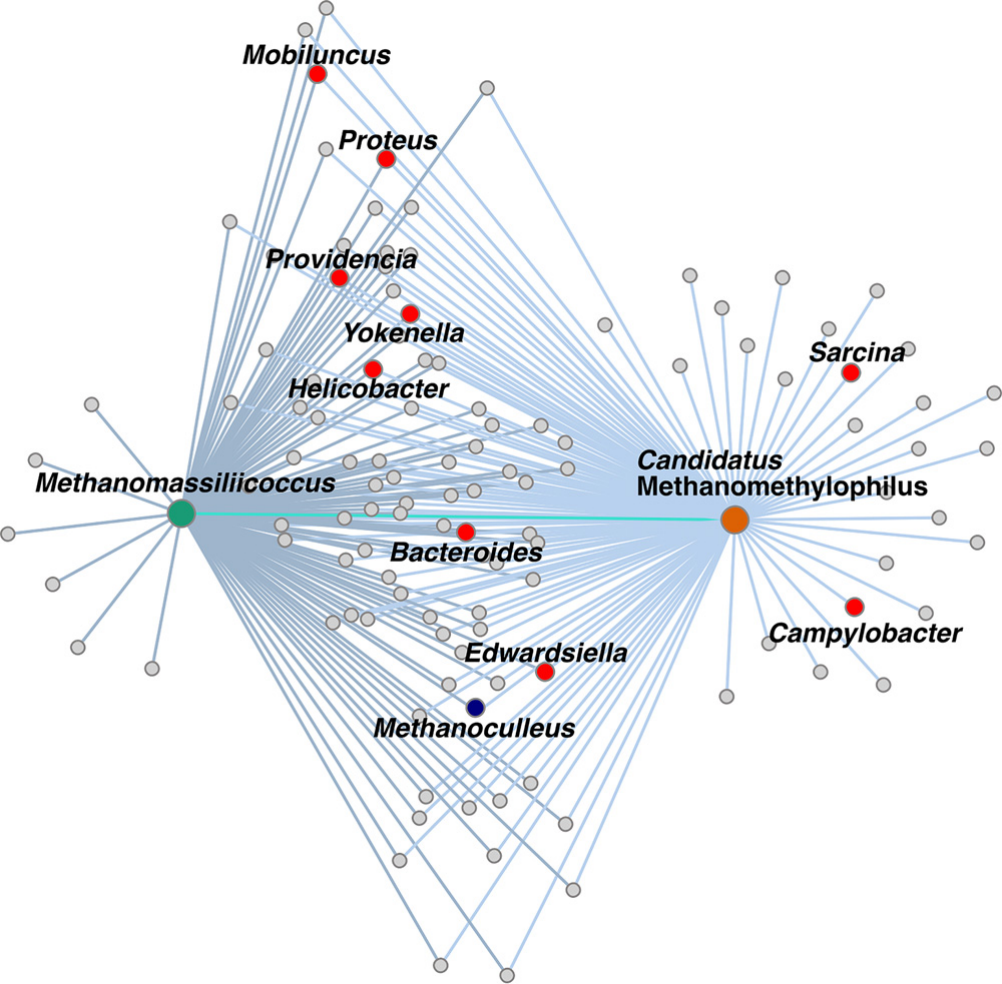
Coabundance networks of *Methanomassiliicoccus* (green node, dark edges) and “*Ca*. Methanomethylophilus” (orange node, light edges) in the human gut largely overlap. Both *Methanomassiliicoccales* genera are significantly coabundant (cyan edge). Their abundances are also coordinated with those of another archaeon (blue node) and TMA-producing bacterial taxa (red nodes).

Analysis of the combined network of “*Ca*. Methanomethylophilus” and *Methanomassiliicoccus* revealed a large overlap between taxa associated with either genus (Fig. 6 and Table S1F): out of 119 taxa in the network, 86 (72.3%) were associated with both. Moreover, 51 taxa (42.9%) also had a significant positive cooccurrence pattern with both genera (adj. *P* < 0.05 in all cases). Most bacterial members of this network had low relative abundances; only *Bacteroides* and *Parabacteroides* had a mean relative abundance above 1% (range, 22.7% to 0.0005%). Interestingly, they included taxa that can potentially produce TMA, since their genomes contain genes encoding enzymes involved in its synthesis; these taxa included Bacteroides, Campylobacter, Yokenella, Mobiluncus, Proteus, Providencia, and Edwardsiella (27).

### Abundance of *Methanomassiliicoccales* species is not concordant in monozygotic or dizygotic human twins.

To evaluate whether host genetics influences the abundance of *Methanomassiliicoccales* in the human gut, we compared the intraclass correlation coefficient (ICC) of their abundances at the genus level using a set of 153 monozygotic (MZ) and 200 dizygotic (DZ) twin pairs from the TwinsUK cohort. As a control, we first compared the mean ICC across all taxa between MZ and DZ twins and found that ICC_MZ_ (0.1) was significantly higher than ICC_DZ_ (0.03) (*P* < 0.01). In addition, we assessed the ICC values of bacterial (Christensenella, Faecalibacterium, and Bifidobacterium) and archaeal (*Methanobrevibacter*) genera, and consistently found a higher correlation for MZ compared to DZ twins (Table S1G). We were only able to assess ICC values of *Methanomassiliicoccus*, as it was the only *Methanomassiliicoccales* taxon detected in the twins with a prevalence (8.64%) above the 5% cutoff (see Materials and Methods). We did not detect a significant concordance between the abundances of *Methanomassiliicoccus* in MZ (ICC_MZ_ = 0.004; adj. *P* = 0.59) or in DZ twins (ICC_DZ_ = 0.017; adj. *P* = 0.71). Given the low abundance of *Methanomassiliicoccales* taxa, we performed a sensitivity analysis using samples with a high sequencing depth (>12 million reads/sample); however, we did not observe differences in the abundance and prevalence of the *Methanomassiliicoccales* genera or in the ICC estimates (data not shown).

## DISCUSSION

While the source of the members of the *Methanomassiliicoccales* has been noted in previous surveys of single markers such as 16S rRNA and *mcrA* genes (10, 11), here, we searched metagenomes from host-associated and environmental samples for their relative abundances. Overall, the HA taxa were enriched in host-associated samples and the FL taxa were enriched in environmental samples; intriguingly, all taxa, regardless of clade, were detected in both biomes. This suggests that members of the order *Methanomassiliicoccales* are generalists with an overall habitat preference according to clade, although there were some exceptions to the general pattern. We show that members of *Methanomassiliicoccales* use many of the same adaptations to the gut as other methanogens. These adaptations include genome reduction and genes involved in the shikimate pathway and bile resistance. In addition, gut-enriched taxa tend to have a distinct repertoire of genes encoding adhesion factors. We observed that potential adaptations to the gut differed by clade, not preferred habitat, indicating convergence on a shared niche through different genomic solutions. In the human gut, *Methanomassiliicoccales* taxa correlated with TMA-producing bacteria, rather than host genetics or other host factors.

For members of the HA clade, adaptations to life in the gut included an enrichment of genes involved in bile acid transport, efflux pumps, and hydrolases, which play a role in tolerance to these compounds in the gastrointestinal tract (28). This adaptation is also shared with other members of the gut microbiota, including *Methanobacteriales* taxa; Methanobrevibacter smithii and Methanosphaera stadtmanae are resistant to bile salts (3, 4). Other gene clusters with known functions enriched in clade HA are involved in metabolism of shikimate and chorismate. The shikimate pathway is involved in the synthesis of aromatic amino acids in plants and microbes, but it is absent in mammals. Shikimate metabolism is carried out by archaeal (29) and bacterial (30, 31) members of the animal gut microbiota and was reported as one of the most conserved metabolic modules in a large-scale gene catalogue from the human gut (32). In turn, aromatic amino acids can be transformed by the gut microbiota into active metabolites, which are involved in diverse physiological processes (33) and conditions such as cardiovascular disease (34). Indeed, plasma concentrations of microbial derivatives of tryptophan have even been shown to negatively correlate with atherosclerosis (35). It remains to be elucidated whether *Methanomassiliicoccales* are involved in human health through the metabolism of aromatic amino acids and associated compounds.

We observed that each clade tended to encode different adhesion factors, although without statistical significance. These factors are involved in the maintenance of syntrophic relationships of the methanogens with bacterial (12, 36) or eukaryotic (37) microorganisms. Two groups of adhesion factors, proteins containing Sel1 domains and *Listeria-Bacteroides* repeats, have been previously studied in *Methanomassiliicoccales* taxa retrieved from the gut (9, 23). Our assessment of these factors in the broader context of the order *Methanomassiliicoccales* showed that these two groups are more likely to be higher in clade HA than clade FL taxa, with the exception of the outlier taxa. Indeed, the repertoire of ELPs and ALPs was similar between species inhabiting the gut, regardless of their clade. This emphasizes the potential involvement of these proteins in the adaptation to the intestinal environment, although the exact mechanisms are yet to be elucidated.

In contrast, members of clade FL appear to be generalists that colonized the animal gut independently from the HA clade. It has been previously noted that *M. luminyensis*, an outlier from clade FL, could have a facultative association to the animal gut. It possesses genes involved in nitrogen fixation, oxidative stress (9), and mercury methylation (23), which are common in soil microorganisms but rare in members of the gut microbiota (38). In accordance with this, we observed that members of clade FL are widespread and abundant in soil, water, and gut metagenomes, with a preference for environmental biomes. Similarities in ELP content between gut-dwelling taxa from both clades indicate that interaction with the host or other members of the gut microbiota might be a key factor in the adaptation of these methanogens.

Analysis of the gene content of outlier taxa from clade FL showed that they tended to be more similar to members of their own clade than to taxa from clade HA, with the exception of “*Ca*. M. intestinalis” Issoire-Mx1, which was distinct from either clade FL and HA. In addition, there was little overlap in gene clusters commonly observed in clade HA and outlier taxa from clade FL, with the exception of the adhesion factors discussed above. These observations support the hypothesis that colonization of animal guts by members of *Methanomassiliicoccales* occurred in two independent events (9, 23), and suggests that there is not one solution to life in the gut for these archaea, as members from two clades seem to have solved the problem with a different set of adaptations.

Characterization of the abundance of *Methanomassiliicoccales* taxa across human populations showed members of this group are rare in the microbiota of healthy adults. We did not detect them in all the studied populations, and, when detected, they had low prevalence and abundance. Our extensive analysis of human gut samples corroborates estimates of *Methanomassiliicoccales* prevalence (up to 11%) (23, 39, 40) and mean abundance (below 1%) (23, 41). Differences in *Methanomassiliicoccales* carriage between Westernized and non-Westernized populations remain to be explained, and may be due to diet. While Westernized diets are richer in TMA precursors than non-Western diets (42), intake varies across populations (43).

Our analysis allowed us to assess whether *Archaea* in the human gut are mutually exclusive. We observed positive correlations of “*Ca*. Methanomethylophilus” and *Methanomassiliicoccus* with each other and with *Methanoculleus*, another rare archaeal member of the gut microbiota (45). We did not find evidence of association between members of *Methanomassiliicoccales* and *Methanobrevibacter*, positive or otherwise, confirming the previous report that these methanogens are not mutually exclusive (39); abundance of H_2_ in the gut, together with differences in other substrate utilization, might result in nonoverlapping niches (46).

While genus *Methanobrevibacter* was consistently found to have a moderate heritability in the TwinsUK (19, 39, 47) and other cohorts (48, 49), this was not the case for members of *Methanomassiliicoccales*. Similarly to humans, methane production (50) and abundance of *Methanobrevibacter* (51) are also heritable in bovine cattle, but *Methanomassiliicoccales* taxa are not (51). Thus, host genetics might be linked to particular taxa and methanogenesis pathways, not to all *Archaea* or to methane production as a whole.

Genera “*Ca*. Methanomethylophilus” and *Methanomassiliicoccus* cooccur with TMA-producing bacteria (27), further supporting their potential use as a way of targeting intestinal TMA (52). The exact nature of the ecological relationships each of these taxa establishes with other members of the microbiome remains to be elucidated. In a facilitation scenario between the methanogens and H_2_ and TMA producers, freely available TMA and H_2_ required for methylotrophic methanogenesis could be utilized by *Methanomassiliicoccales* taxa (53) without cost to the producer. Alternatively, the methanogens could establish syntrophic interactions with other microorganisms, whereby the consumption of these metabolites is also beneficial to the producer (53).

The present study extends our understanding of the order *Methanomassiliicoccales* by revealing genomic adaptations to life in the gut by members of both clades that make up this group. Furthermore, the positive correlation between the relative abundances of these TMA-utilizing *Archaea* with TMA-producing bacteria in the gut is a first step toward understanding how they may be harnessed for therapeutic management of gut TMA levels in the context of cardiovascular disease.

## MATERIALS AND METHODS

For a detailed description of the methods, see Text S1 in the supplemental material.

### Genome annotation and phylogenomic tree reconstruction.

We used 71 substantially complete genomes (completeness, ≥70%) with low contamination (contamination, <5%) retrieved from the NCBI Assembly database (https://www.ncbi.nlm.nih.gov/assembly), plus an additional high-quality metagenome-assembled genome (MAG) corresponding to “*Candidatus* Methanomethylophilus alvus.” Gene calling was performed using Prokka (54). A maximum-likelihood phylogenomic tree was constructed using PhyloPhlAn (55) with the 72 *Methanomassiliicoccales* genomes plus members of the order Thermoplasmatales as an outgroup. We used interactive Tree Of Life (iTOL) (56) to visualize the tree.

### Abundance of *Methanomassiliicoccales* in environmental and animal gastrointestinal metagenomes.

We retrieved 305 publicly available gastrointestinal and environmental metagenome samples (57) (see Table S1B in the supplemental material). To avoid multiple mapping of reads, we dereplicated the 72 genomes at a species level (95% average nucleotide identity [ANI]) using dRep (58), resulting in 29 representative genomes. We quantified the abundance of dereplicated *Methanomassiliicoccales* genomes in the metagenomes using KrakenUniq (59). We estimated the enrichment of each representative *Methanomassiliicoccales* taxon in host or environmental metagenomes using DESeq2 with the Wald test (60) on sequence counts and classifying metagenome samples as either host derived or environmental.

### Comparative genomics.

We grouped the predicted genes into gene clusters using panX (61) and used InterProScan (62) and eggNOG-mapper (63) for annotation. Phylogenetic signal of genome characteristics and gene cluster presence was tested using the *phylosignal* R package with the local indicator of phylogenetic association (LIPA) (64). The R package *micropan* (65) was used to create a pangenome principal-component analysis (PCA). We performed phylogenetic ANOVA using the R package *phytools* (66) to determine clusters enriched in clades FL or HA. We adjusted *P* values for multiple comparisons with the Benjamini-Hochberg method. Due to the exploratory nature of this work, tests were considered significant if they had an adjusted *P* value (adj. P) of <0.1; a false-discovery-rate-adjusted *P* value cutoff of 0.1 implies that 10% of significant tests will result in false-positives. In cases where adjusting *P* values was not necessary, raw *P* values are provided.

We assessed the presence of eukaryote-like proteins (ELPs) (24) by combining the counts of gene clusters classified as Sel1-containing proteins (Sel1), *Listeria-Bacteroides* repeat-containing proteins (List-Bact), tetratricopeptide repeats (TPR), ankyrin repeats (ANK), leucine-rich repeats (LRR), fibronectin type III (FN3) domains, laminin G domains, bacterial Ig-like domains, *Yersinia* adhesin A-like domain (YadA), TadE-like domain, or invasion protein B (IalB). Likewise, we characterized the presence of parallel beta-helix-repeat-containing proteins, also known as adhesin-like proteins (ALPs).

### Characterization of *Methanomassiliicoccales* distribution across human populations.

We retrieved and quality controlled 4,472 publicly available human gut metagenomes from 34 independent studies (Table S1C). Reads were classified using Kraken (67) and Bracken (68) with custom databases (69). Taxa with <100 reads in a given sample were considered absent. To determine the cooccurrence patterns of *Methanomassiliicoccales* in the human gut, we used the *cooccur* package (26); to determine their coabundance patterns, we calculated the proportionality of taxa abundance (rho) with the *propr* R package (70). The *lme4* and *lmerTest* R packages (71) were used to fit linear mixed effects models to test differences of *Methanomassiliicoccales* genera abundance by Westernization status, age, and gender. We employed binomial linear mixed models to test differences in genera prevalence. Lists of potential TMA (27, 72, 73) and methanol (74) (see Text S1 in the supplemental materials) producers were compiled from the available literature.

Heritability of *Methanomassiliicoccales* taxa was assessed by comparing relative abundances of taxa within 153 monozygotic (MZ) and 200 dizygotic (DZ) twin pairs from the United Kingdom Adult Twin Registry (TwinsUK) (19, 39, 75). Absolute read counts were transformed using the Yeo-Johnson transformation and adjusted by body mass index (BMI), sex, and sequencing depth (19, 39). We calculated the intraclass correlation coefficient (ICC) in MZ and DZ twins with the *irr* R package, and adjusted *P* values using the Benjamini-Hochberg method. We compared the mean ICC across all taxa between MZ and DZ twins using the Mann-Whitney test and by assessing the ICC of taxa previously reported as heritable (*Methanobrevibacter*, *Faecalibacterium*, *Christensenella*, and Bifidobacterium) (39, 48).

### Data availability.

The raw sequence data are available from the European Nucleotide Archive under study accession number PRJEB40256. Jupyter notebooks are available at https://github.com/leylabmpi/Methanomassilii. The “*Candidatus* Methanomethylophilus” MAG generated here can be found at http://ftp.tue.mpg.de/ebio/projects/Mmassilii/.
