# Supplementary material for: Genomic Insights into Adaptations of Trimethylamine-Utilizing Methanogens to Diverse Habitats, Including the Human Gut
Source: mSystems. 2021 Feb 9;6(1):e00939-20. doi: 10.1128/mSystems.00939-20 (PMC7883539; doi:10.1128/mSystems.00939-20)
Supplement: TEXT S1 [file mSystems.00939-20-s0001.docx]

Supplementary Methods

# Sample preparation, DNA extraction and shotgun metagenome sequencing

Total DNA was extracted from each of 128 human stool samples previously collected (1) using the PowerSoil® - htp DNA isolation kit (MoBio Laboratories Ltd, Carlsbad, CA). We constructed metagenome libraries using a Nextera protocol modified to include smaller volumes (2). After purification with Agencourt AMPure XP beads (Beckman Coulter), samples were normalized and pooled. We then performed size selection of the pooled samples using BluePippin (Sage Sciences) to restrict fragment sizes between 300 to 650 bp. Pools were sequenced using the Illumina HiSeq 3000 platform with 2x150 bp paired end sequencing.

#

# Sequence quality control

We validated raw reads with fqtools v.2.0 (3), de-duplicated reads with the clumpify module of bbtools v.37.78 (https://jgi.doe.gov/data-and-tools/bbtools/), and carried out adapter trimming and read quality control with skewer v.0.2.2 (4) and the bbduk module of bbtools. Next, we filtered reads that mapped to the hg19 assembly using the bbmap module of bbtools. We obtained QC reports for all reads with fastqc v.0.11.7 (https://github.com/s-andrews/FastQC) and multiQC v.1.5a (5). Publicly available samples downloaded from the sequence read archive (SRA) were processed using the same workflow with exception of the clumpify module of bbtools.

# Metagenome assembly

We performed a co-assembly using megahit v.1.1.2 (6). The co-assembly was assessed using MetaQuast v.4.6.3 (7). Next, we used bowtie2 v.2.2.5 to map the reads of each sample to the assembled contigs and binned the contigs using binsanity v.0.2.6 (8), maxbin2 v.2.2.2 (8,9) (ran twice with the following parameters: -prob_threshold 0.6, or -prob_threshold 0.8), and metabat v.2.12.1 (10) (ran twice with the following parameters: --maxP 92 --maxEdges 150, or --maxP 97 --maxEdges 500). Finally, we used DAS-Tool v.1.1.0 (11) to score and dereplicate bins, and the retained bins were were quality-assessed with CheckM v.1.0.7 (12) and sourmash v.2.0.0 (13).

# Quality control and taxonomic classification of the metagenome-assembled genome

We extracted 16S rRNA gene sequences from the metagenome-assembled genome (MAG) using Barnap v.0.9 (https://github.com/tseemann/barrnap) and used BLASTn to classify the 16S rRNA gene sequences from the MAG using the SILVA 132 database clustered at 97 % (https://www.arb-silva.de/download/archive/qiime). All sequences from SILVA that had a sequence identity ≥97 % to the 16S rRNA gene from the MAG belonged to the genus “*Candidatus* Methanomethylophilus”. We confirmed the classification of the assembled genome as a member of the genus “*Ca.* Methanomethylophilus” by using a set of *Methanomasiliicoccales* genomes (see *comparative genomics* below) to calculate the average nucleotide identity (ANI) using dRep v.2.0.5 (14). In addition, we verified the phylogenetic origin of the predicted genes of the obtained assembly using MyTaxa scan (15) as available in the Microbial Genomes Atlas (MiGA) (16).

# Genome annotation and phylogenomic tree reconstruction

We downloaded 78 available genomes belonging to the order *Methanomassiliicoccales* from the NCBI assembly database (https://www.ncbi.nlm.nih.gov/assembly) as available in June 2018, and used CheckM to assess their quality. For subsequent analyses, we included 71 substantially complete genomes (completeness ≥70%) with low contamination (contamination <5%) (20), plus an additional high-quality metagenome-assembled genome (MAG) corresponding to “*Candidatus* Methanomethylophilus alvus” (table S1A). On average, the included genomes had a high completeness (mean ± sd: 91.6%±5.7) and low contamination (0.6%±0.8). Gene calling, proteome prediction and annotation was performed on each genome using Prokka 1.12 (21). Details of each genome, including the original source of isolation, can be found in (table S1A).

Using PhyloPhlAn 0.26 (22), we constructed a maximum-likelihood phylogenomic tree using a concatenated alignment of multiple universally distributed single copy marker genes of the 72 included genomes from the order *Methanomassiliicoccales* (71 publicly available plus our assembled “*Ca.* M. alvus” MAG). Of these, one was retrieved from pure culture, 6 were obtained from enrichment cultures and 64 were MAGs. We included an additional MAG retrieved from human gut metagenomes corresponding to “*Ca*. M. alvus” (*supplementary results*). Briefly, universal markers were obtained from the translated amino acid sequences of the included genomes, aligned using mafft 7.3 (23) and concatenated into a single sequence. We then used the concatenated alignment to reconstruct an maximum-likelihood phylogenetic tree using RAxML 8.1 (24); branch support was estimated by 1000 bootstrap iterations and the tree was rooted by including members of the order *Thermoplasmatales* as outgroup, namely *Thermoplasma acidophilum* DSM 1728 (GenBank assembly accession: GCA_000195915.1), *Picrophilus oshimae* DSM 9789 (GCA_900176435.1), *Ferroplasma acidarmanus* fer1 (GCA_000152265.2), *Acidiplasma aeolicum* (GCA_001402945.1) and *Cuniculiplasma divulgatum* (GCA_900090055.1). We used iTOL (25) to visualize the tree.

# Abundance of Methanomassiliicoccales in environmental and animal gastrointestinal metagenomes

We retrieved 305 metagenome samples of gastrointestinal and environmental origin (26) sequenced using the Illumina HiSeq platform (table S1B). Sequences were then downloaded from the Sequence Read Archive (SRA) and quality-controlled. To avoid the issue of multiple mapping, we dereplicated the 72 genomes at a species-level threshold (95% ANI) using dRep, resulting in 29 representative genomes. Next, we quantified the abundance of dereplicated *Methanomassiliicoccales* genomes in these samples using KrakenUniq v.0.5.8 (27), which maps sequence reads to a database containing the 29 representative genomes. A representative genome was considered present in a sample if at least 100 unique k-mers were detected. Statistical analyses were performed using R v.3.5.1 (28). We estimated the enrichment of each representative *Methanomassiliicoccales* on host or environmental metagenomes using DESeq2 v.1.22 with the Wald test (29) on sequence counts and classifying metagenome samples as either host-derived or environmental. We applied hierarchical clustering using Ward’s method on the log-fold-change of environmental vs gastrointestinal enrichment of each taxon and calculated the cophenetic correlation with the phylogenomic tree using the ape v.5.3 package of R (30).

# Comparative genomics

The predicted proteome of each included genome was used to to assign orthology clusters using panX 1.6.0 (31). We used InterProScan (32) and eggNOG mapper 1.0.3 (33) with DIAMOND 0.8.36 (34) against the optimized archaeal database to improve the annotation of gene clusters. Phylogenetic signal of genome characteristics and gene cluster presence was tested using the phylosignal v.1.2 package of R with the local indicator of phylogenetic association (LIPA) (35).

The R package micropan v.2.0 (36) was used to create a principal component analysis (PCA) of gene cluster presence. We compared the gene cluster content between clades to determine gene clusters enriched on clades FL or HA using phylogenetic ANOVA using the R package phytools v.0.7 (37). To reduce the number of comparisons we first removed low frequency and other non-informative gene clusters by filtering those with near zero variance using the caret v.6.0 package of R, that is, gene clusters with counts that are largely constant across all the genomes (*e.g.* singleton and doubleton gene clusters). We reasoned that very rare gene clusters present in few genomes, or clusters that don’t vary across the phylogeny are not informative to determine differences in the adaptation to the environment. The above analysis was repeated by comparing gene cluster content between taxa significantly enriched on gut or environmental samples, prior removal of taxa not significantly enriched in either biome class. We adjusted P values for multiple comparisons with the Benjamini-Hochberg method; tests were considered significant if they had an adjusted P value (adj. P) < 0.1. In cases where adjusting P values was not necessary, raw P values (P val.) are provided.

We assessed the presence of eukaryote-like proteins (ELPs) (38) by combining the counts of gene clusters classified by InterProScan as any of the following: Sel1 containing proteins (Sel1), Listeria-Bacteroides repeat containing proteins (List-Bact), tetratricopeptide repeats (TPR), Ankyrin repeats (ANK), Leucine-rich repeats (LRR), Fibronectin type III (FN3) domains, Laminin G domain, Bacterial Ig-like domains, Yersinia adhesin A-like domain (YadA), TadE-like domain or Invasion protein B (ialB). Likewise, we characterized the presence of parallel beta-helix repeat-containing proteins, also known as adhesin-like proteins (ALPs).

# Characterization of Methanomassiliicoccales distribution across human populations

We obtained sample metadata from publicly available studies using the curatedMetagenomicData v.1.17 package of Bioconductor (39). Samples were selected according to the following criteria: i) shotgun gut metagenomes sequenced using the Illumina HiSeq platform with a median read length > 95 bp; ii) with available SRA accession; iii) labeled as adults or seniors, or with a reported age ≥ 18 years; iv) without report of antibiotic consumption (i.e. no or NA); v) without report of pregnancy (i.e. no or NA); vi) non-lactating women (i.e. no or NA); vii) without report of gangrene, pneumonia, cellulitis, adenoma, colorectal cancer, arthritis, Behcet's disease, cirrhosis or inflammatory bowel disease. These criteria allowed the inclusion of subjects with conditions such as obesity and type 2 diabetes. While these are known to be associated with changes in the composition of the microbiome, said changes are relatively minor compared to excluded diseases.

Since not all available samples had paired, we only used forward reads to maximize the number of samples used processed in a uniform fashion. A total of 4472 samples from 34 independent studies were downloaded from the SRA between December 2019 and February 2020 (table S1C) and quality controlled as described above.

Reads were classified using Kraken v.2.0 (40) and a Bayesian re-estimation of the species-level abundance of each sample was then performed using Bracken v.2.2 (41). We utilized custom databases created using the Struo pipeline (42) based on GTDB release 86 (available at http://ftp.tue.mpg.de/ebio/projects/struo/). Taxa with <100 reads in a given sample were considered as absent. We obtained complete taxonomic annotations from NCBI taxIDs with TaxonKit 0.2.4 (https://bioinf.shenwei.me/taxonkit/). To determine the cooccurrence patterns of the detected *Methanomassiliicoccales* in the human gut we used the cooccur v.1.3 package of R (43); to determine their coabundance patterns, we calculated the proportionality of taxa abundance (*rho*) with the propr v.4.2 package (44). The lme4 v.1.1 and lmerTest v.3.1 R packages (45) were used to fit linear mixed effects models to test differences of *Methanomassiliicoccales* genera log-transformed abundance by westernization status, age and gender with F-tests and P-values determined via the Satterthwaite's method (ANOVA Type II sum of squares). Similarly, we employed binomial linear mixed models to test differences of *Methanomassiliicoccales* genera prevalence. A list of potential TMA producers was compiled from the available literature (46–48)

We assessed the heritability of *Methanomassiliicoccales* taxa by comparing relative abundances within 153 monozygotic (MZ) and 200 dizygotic (DZ) twin pairs using the taxonomic profiles of 706 gut metagenome samples from the United Kingdom Adult Twin Registry (TwinsUK) (19, 49, 50) with a sequencing depth >5 million reads/sample. We aggregated abundances at the genus level and removed genera with a prevalence <5%. Absolute read counts were transformed using the Yeo-Johnson transformation and adjusted by body mass index (BMI), sex and sequencing depth (19, 49). For each genus, we calculated the intraclass correlation coefficient (ICC) in MZ and DZ twins with the irr v.0.84 package of R, and adjusted P-values for multiple comparisons using the Benjamini-Hochberg method. As control we compared the mean ICC across all taxa between MZ and DZ twins using the Mann-Whitney test, and by assessing the ICC of specific taxa known previously reported as heritable in the same population (*Methanobrevibacter*, *Faecalibacterium*, *Christensenella* and *Bifidobacterium*) (49, 51). We carried a sensitivity analysis by repeating these analyses on a subset of 394 samples (80 MZ and 117 DZ twin pairs) with a sequencing depth of >12 million reads/sample.

Supplementary Results

# A high-quality genome from “Candidatus Methanomethylophilus alvus” was retrieved from TwinsUK metagenomes

We assembled 128 human gut metagenome samples of adult subjects from the TwinsUK cohort and obtained a total of 317 metagenome assembled genomes (MAGs), of which 115 were of high quality (≥90 % completeness, <5 % contamination). Two MAGs were classified as *Archaea* by sourmash; one corresponded to *M. smithii* and the other was only classified at the phylum level. Two partial 16S rRNA gene sequences of the unclassified MAG consistently matched “*Candidatus* Methanomethylophilus” with identity ≥97 % when queried against the SILVA database v.132, and also matched the unclassified *Methanomassiliicoccales* OTU from (1). The MAG had an average nucleotide identity (ANI) of 98.9 % to the genome of “*Candidatus* Methanomethylophilus alvus” Mx1201.

The assembly was composed of 48 contigs with a total length of 1.59 Mb and a GC content of 55.9 %. It was near complete with no contamination, and inspection of MyTaxa scan results did not reveal horizontally transferred regions, contamination or erroneous assembly (table S1A). We detected 1 copy of the 23S rRNA gene, 2 partial 16S rRNA gene copies, and 3 copies of the 5S rRNA gene. We also detected 39 tRNA genes, one of them corresponding to non-standard amino acid pyrrolysine, which is present in monomethylamine, dimethylamine, and trimethylamine:corrinoid methyltransferases needed for methylotrophic methanogenesis, among other proteins (17). A total of 1575 coding sequences were predicted, of which 794 had no annotation.

# Core genes functions differ between Methanomassiliicoccales clades

We defined the soft core genome of a clade as the set of clusters present in >80 % of its member taxa, regardless of their presence in other clades, and compared the composition of the gene clusters in broad functional terms given by the COG categories. The proportion of clusters of unknown functions in the core genome of each clade was large and varied between clades, ranging from 23.0 % in Clade HA to 38.5 % in Clade EX (figure S2 C). The proportion of unknown clusters was lowest in the complete taxonomic order, where it only accounted for 14.7 % of gene clusters.

We also observed differences in the proportion of several functional categories in the core genomes of each of the clades by phylogenetic ANOVA after adjusting P values for multiple comparisons using the Benjamini-Hochberg method (Adj. P < 0.05 in all cases): the core of Clade FL was enriched in gene clusters involved in energy production and conversion (C); cell cycle control, cell division, chromosome partitioning (D); cell wall/membrane/envelope biogenesis (M). Members of the core of Clade HA had the highest proportion of gene clusters belonging to the coenzyme transport and metabolism (H); translation, ribosomal structure and biogenesis (J); and replication, recombination and repair (L). Likewise, Clade EX had the highest proportion of clusters relating to lipid transport and metabolism (I); cell motility (N); secondary metabolites biosynthesis, transport, and catabolism (Q) ;signal transduction mechanisms (T); and cytoskeleton (Z) (figure S2 C). Conversely, the proportions of gene clusters involved in amino acid transport and metabolism (E); nucleotide transport and metabolism (F); carbohydrate transport and metabolism (G); transcription (K); post-translational modification, protein turnover, and chaperones (O); inorganic ion transport and metabolism (P); and defense mechanisms (V) did not differ significantly between clades (Adj. P > 0.1 in all cases).

***Methanomassiliicoccales* are not associated with methanol producers in the human gut**

We looked into the presence of methanol producers in the association networks of *Methanomassiliicoccus* and “*Ca.* Methanomethylophylus” in the human gut, similarly to the analysis performed with TMA-producing taxa. We obtained a list of the most abundant bacteria that carry genes encoding methanol-producing enzymes in stool samples, namely, the genera *Eubacterium*, *Escherichia*, *Parabacteroides*, *Bifidobacterium*, *Butyrivibrio*, *Bacteroides*, *Ruminococcus*, *Clostridium*, *Geobacillus* and *Alistipes* (1). The abundances of *Bacteroides* and *Parabacteroides* were moderately associated with *Methanomassiliicoccus* (rho = 1.8 and 1.4, respectively). Similarly, "*Ca.* Methanomethylophilus" was moderately correlated with *Bacteroides* (rho = 1.2) (table S1F). Note, however, that *Bacteroides* is also one of the potential TMA-producing taxa. This results does not necessarily imply that *Methanomassiliicoccales* are not involved in the metabolism of methanol in the human gut. Rather, if they are, the source of this substrate might not be bacterial but dietary, as fruits and vegetables are the main source of methanol in humans (1).

References

1. Goodrich JKK, Waters JLL, Poole ACC, Sutter JLL, Koren O, Blekhman R, et al. Human Genetics Shape the Gut Microbiome. Cell [Internet]. 2014 Nov;159(4):789–99. Available from: http://dx.doi.org/10.1016/j.cell.2014.09.053

2. Karasov TL, Almario J, Friedemann C, Ding W, Giolai M, Heavens D, et al. Arabidopsis thaliana and Pseudomonas Pathogens Exhibit Stable Associations over Evolutionary Timescales. Cell Host Microbe [Internet]. 2018 Jul 11;24(1):168–79.e4. Available from: http://dx.doi.org/10.1016/j.chom.2018.06.011

3. Droop AP. fqtools: an efficient software suite for modern FASTQ file manipulation. Bioinformatics [Internet]. 2016 Jun 15;32(12):1883–4. Available from: http://dx.doi.org/10.1093/bioinformatics/btw088

4. Jiang H, Lei R, Ding S-W, Zhu S. Skewer: a fast and accurate adapter trimmer for next-generation sequencing paired-end reads. BMC Bioinformatics [Internet]. 2014 Jun 12;15:182. Available from: http://dx.doi.org/10.1186/1471-2105-15-182

5. Ewels P, Magnusson M, Lundin S, Käller M. MultiQC: summarize analysis results for multiple tools and samples in a single report. Bioinformatics [Internet]. 2016 Oct 1;32(19):3047–8. Available from: http://dx.doi.org/10.1093/bioinformatics/btw354

6. Li D, Luo R, Liu C-M, Leung C-M, Ting H-F, Sadakane K, et al. MEGAHIT v1.0: A fast and scalable metagenome assembler driven by advanced methodologies and community practices. Methods [Internet]. 2016 Jun 1;102:3–11. Available from: http://dx.doi.org/10.1016/j.ymeth.2016.02.020

7. Mikheenko A, Saveliev V, Gurevich A. MetaQUAST: evaluation of metagenome assemblies. Bioinformatics [Internet]. 2016 Apr 1;32(7):1088–90. Available from: http://dx.doi.org/10.1093/bioinformatics/btv697

8. Graham ED, Heidelberg JF, Tully BJ. BinSanity: unsupervised clustering of environmental microbial assemblies using coverage and affinity propagation. PeerJ [Internet]. 2017 Mar 8;5:e3035. Available from: http://dx.doi.org/10.7717/peerj.3035

9. Wu Y-W, Simmons BA, Singer SW. MaxBin 2.0: an automated binning algorithm to recover genomes from multiple metagenomic datasets. Bioinformatics [Internet]. 2016 Feb 15;32(4):605–7. Available from: http://dx.doi.org/10.1093/bioinformatics/btv638

10. Kang DD, Froula J, Egan R, Wang Z. MetaBAT, an efficient tool for accurately reconstructing single genomes from complex microbial communities. PeerJ [Internet]. 2015 Aug 27;3:e1165. Available from: http://dx.doi.org/10.7717/peerj.1165

11. Sieber CMK, Probst AJ, Sharrar A, Thomas BC, Hess M, Tringe SG, et al. Recovery of genomes from metagenomes via a dereplication, aggregation and scoring strategy. Nat Microbiol [Internet]. 2018 Jul;3(7):836–43. Available from: http://dx.doi.org/10.1038/s41564-018-0171-1

12. Parks DH, Imelfort M, Skennerton CT, Hugenholtz P, Tyson GW. CheckM: assessing the quality of microbial genomes recovered from isolates, single cells, and metagenomes. Genome Res [Internet]. 2015 Jul;25(7):1043–55. Available from: http://dx.doi.org/10.1101/gr.186072.114

13. Titus Brown C, Irber L. sourmash: a library for MinHash sketching of DNA. JOSS [Internet]. 2016 Sep 14;1(5):27. Available from: http://joss.theoj.org/papers/10.21105/joss.00027

14. Olm MR, Brown CT, Brooks B, Banfield JF. dRep: a tool for fast and accurate genomic comparisons that enables improved genome recovery from metagenomes through de-replication. ISME J [Internet]. 2017 Dec;11(12):2864–8. Available from: http://dx.doi.org/10.1038/ismej.2017.126

15. Luo C, Rodriguez-R LM, Konstantinidis KT. MyTaxa: an advanced taxonomic classifier for genomic and metagenomic sequences. Nucleic Acids Res [Internet]. 2014 Apr;42(8):e73. Available from: http://dx.doi.org/10.1093/nar/gku169

16. Rodriguez-R LM, Gunturu S, Harvey WT, Rosselló-Mora R, Tiedje JM, Cole JR, et al. The Microbial Genomes Atlas (MiGA) webserver: taxonomic and gene diversity analysis of Archaea and Bacteria at the whole genome level. Nucleic Acids Res [Internet]. 2018 Jul 2;46(W1):W282–8. Available from: http://dx.doi.org/10.1093/nar/gky467

17. Borrel G, Parisot N, Harris HMB, Peyretaillade E, Gaci N, Tottey W, et al. Comparative genomics highlights the unique biology of Methanomassiliicoccales, a Thermoplasmatales-related seventh order of methanogenic archaea that encodes pyrrolysine. BMC Genomics [Internet]. 2014 Aug 13;15:679. Available from: http://dx.doi.org/10.1186/1471-2164-15-679
